# Early increases in plasminogen activator activity following partial hepatectomy in humans

**DOI:** 10.1186/1476-5926-3-11

**Published:** 2004-12-23

**Authors:** David Mangnall, Kirsty Smith, Nigel C Bird, Ali W Majeed

**Affiliations:** 1 Liver Research Group, Division of Clinical Sciences South, K Floor, Royal Hallamshire Hospital, Sheffield S10 2JF, UK

## Abstract

**Background:**

Increases in urokinase-like plasminogen activator (uPA) activity are reported to be amongst the earliest events occurring in remnant liver following partial hepatectomy in rats, and have been proposed as a key component of the regenerative response. Remodelling of the extracellular matrix, conversion of single chain hepatocyte growth factor to the active two-chain form and a possible activation of a mitogenic signalling pathway have all been ascribed to the increased uPA activity. The present study aimed to determine whether similar early increases in uPA activity could be detected in the remnant liver following resection of metastatic tumours in surgical patients.

**Results:**

Eighteen patients undergoing partial hepatectomy for the removal of hepatic metastases secondary to primary colonic tumours were studied. Increased plasminogen activator activity was found in the final liver samples for the group of patients in whom the resection size was at least 50%. For smaller resections, the increased activity was not observed. The increased activity did not correlate with the age of the patient or with the time between the start of resection and the end of the operation. There was, however, a negative correlation between plasminogen activator activity and the time for which blood supply to the liver was clamped.

**Conclusions:**

Our findings are in accordance with those from experimental animal models and show, for the first time, that rapid increases in plasminogen activator activity can occur following similarly large liver resection in humans. Thus, increases in plasminogen activator activity are an early event in the remnant liver following major liver resection in man. Our observations provide support for the contention that increases in plasminogen activators play a key role in the initiation of hepatic regeneration in man.

## Background

Urokinase-like plasminogen activator (uPA), initially recognised by its ability to convert plasminogen to plasmin and to participate in the fibrinolytic cascade, is now considered to have a wider role, which encompasses metastatic invasion by tumour cells and liver regeneration. In regeneration of the liver following partial hepatectomy, uPA has a number of potential roles. These include initiating the remodelling of the extracellular matrix to allow cell division, activation of extra-cellular pro-metalloproteases and the release of the bound single-chain form of hepatocyte growth factor (HGF) from the extracellular matrix (ECM). *In vitro *uPA and tissue-like plasminogen activator (tPA) have been shown to convert single chain inactive HGF into the active two chain form [1] in cultures of hepatocytes. In normal rodent liver, both the inactive and active forms of HGF can be detected, with the predominance of the inactive form [2]. Following partial hepatectomy in the rat there is an early net decrease in the total amount of HGF in the liver, but the relative proportion of the single chain, inactive form, is decreased and the active two-chain form increased [2]. This implies an early proteolytic conversion, possibly mediated by the plasminogen activators. The importance of the uPA-plasminogen system to liver repair has been further demonstrated by the inability of plasminogen deficient animals to form regenerative nodules in response to acute liver injury [3]. As discussed by Mangnall *et al. *[4], uPA may also activate a signalling pathway leading to mitosis of the hepatocyte.

Increases in uPA activity are amongst the earliest documented changes following partial hepatectomy in rats [5]. Raised uPA activity was detected in the remnant liver at one-minute post-hepatectomy and continued to increase for at least one hour, although there were no changes in the total amount of uPA protein detectable by Western blotting. The binding of uPA to the uPA receptor (uPAR) is also associated with an increase in uPA enzymatic activity [6]. In the rat partial hepatectomy model, the increase in uPA activity is thought to be due to an increase in the level of uPAR and subsequent binding and activation of uPA. In the remnant liver, increases in the amount of uPAR have been detected by Western blotting also as early as 1 min post hepatectomy and more clearly at 1 hour. This had decreased by 6 h and was back to basal levels by 24 h [5]. The mechanism underlying these changes remains unclear.

Additional support for a role for uPA in the hepatic regenerative process comes from studies of uPA-deficient (uPA-/-) mice. In these animals, uptake of [^3^H]-thymidine into DNA and mitotic index were reduced by almost half at 44 h post-hepatectomy (the peak time for control mice), suggesting a slower hepatocyte growth response [7]. In a separate study uPA-/-mice were treated with anti-Fas monoclonal antibody to induce extensive hepatocyte apoptosis. Fas (a member of the TNF-receptor superfamily) is present in the inactive state as a monomer, but on binding the appropriate ligand (in this case the antibody) the receptors aggregate and activate apoptosis leading to cell destruction. In these uPA-deficient animals, the regeneration response following anti-Fas treatment was delayed relative to normal control animals [8]. Generation of mature HGF and time of peak levels were delayed in the uPA-/-mice and peak levels of proliferating cell nuclear antigen at 96 h were also delayed relative to controls, which peaked at 48 h. Treatment of the uPA-/-mice with the uPA gene by lipofection reversed these effects. The results support a role for uPA in the generation of mature HGF and in the regeneration after Fas-mediated liver damage.

More recently, studies with uPA or plasminogen deficient mice confirmed the requirement for plasminogen activation in liver regeneration and also showed a need for plasminogen in regeneration-associated hepatic angiogenesis [9]. Collectively, these studies strongly suggest that a very early increase in uPA activity is a key feature of the liver regenerative response in rodents. It is generally assumed that regeneration in the human liver follows a similar course but the relative paucity of studies in humans means that, at present, it is unclear whether a similar role for uPA exists in the regeneration of human liver.

Though not necessarily identical, it is clear from the literature that regeneration in humans and rodents share similar mechanisms. Many of the cytokines and growth factors essential for regeneration in rodents [reviewed in [4]] are also found in increased amounts in the regenerating human liver, implying once more similar mechanisms.

However, clear differences between species do exist; a notable example being the differences in the time at which DNA synthesis peaks in the remnant liver. In rats, this is at about 24 h; in mice, at about 40 h; and in man, at 180–200 h following hepatectomy. In the case of the human studies, this may partially reflect the relatively greater age of the patients since the rate of regeneration slows with age. Such age related effects are less likely in the rodent studies where the timing of hepatocyte entry into DNA synthesis following partial hepatectomy has been shown to be an intrinsic, cell-autonomous, feature [10]. Thus, although the basic mechanisms may be fundamentally similar, there are inherent differences between species (such as the timing of the cell cycle clock) which underscores the need not to assume that all aspects of regeneration operate identically in all mammals.

The unique sensitivity of the human hepatocyte to TRAIL (tumour necrosis factor-related apoptosis-inducing ligand) [11] likewise emphasises the need for caution when extrapolating from rodent liver to human liver.

The vast majority of the literature concerns regeneration in rats and mice and much less information is available from human studies since the opportunity to study liver regeneration in humans is generally limited to units specialising in liver surgery and is necessarily constrained by ethical considerations. Surgical removal of liver metastases affords the opportunity to obtain small samples of liver at the start, time of resection and time of wound closure approximating to the early sampling times in the animal studies. In this vein, the aim of the present study was to determine whether very early increases in uPA activity occur in the remnant liver following resection in man.

## Results

The basal uPA activity associated with the membrane preparations showed a wide patient to patient variation ranging from 4 to 24 nmol/min/mg protein with a mean of 9.94 nmol/min/mg protein (n = 18, SD = 5.06). This variation in basal activity correlated neither with the age of the patient – linear regression analysis gave a slope of -0.03 and correlation analysis gave a Spearman coefficient of -0.097 (*p *= 0.7), nor there was any difference between the values for male patients (mean = 9.14 nmol/min/mg, SD = 3.84, n = 7) and female patients (mean = 10.45, SD = 5.83, n = 11) with a non-significant unpaired Student's *t *test (*p *= 0.61).

**Figure 1:**
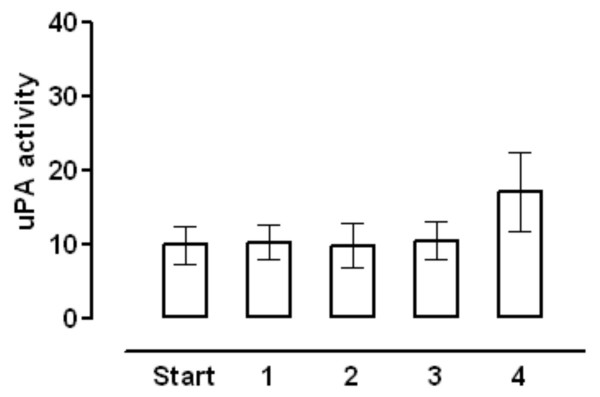
**Membrane associated plasminogen activator activity (nmol/min/mg protein) in the whole patient group. **Samples were: Start (control sample taken at start of operation); 1 = Res 0; 2 = Rem 0; 3 = Res End; and 4 = Rem End, as described in the Methods. Values are means and the error bars are 95% confidence limits. Student's paired *t *test analysis showed the Start and Rem End samples to be statistically significantly different (*p *= 0.01, n = 18). There were no statistical differences between start and any of the other samples.

**Figure 2:**
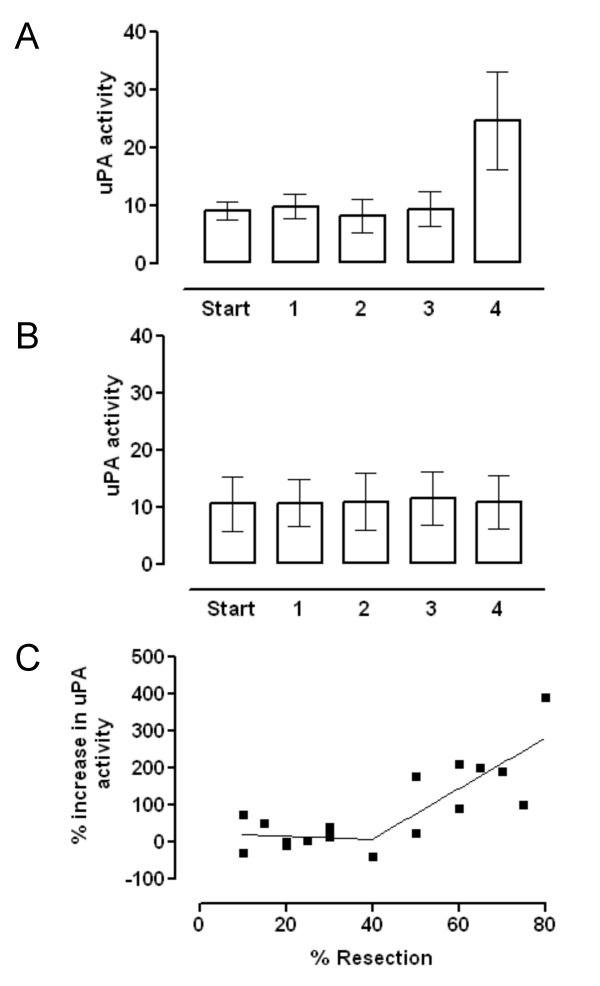
**Membrane associated plasminogen activator activity (nmol/min/mg protein) in the group for whom the estimated resection size was 50% or greater and for the group where the resection size was less than 50%. **Samples were: Start (control sample taken at start of operation); 1 = Res 0; 2 = Rem 0; 3 = Res End; and 4 = Rem End; as described in the Methods. Values are means and the error bars are 95% confidence limits. For the 50% and greater group (Figure 2A), Student's paired *t *test analysis showed the Start and Rem End samples to be statistically significantly different (*p *= 0.002, n = 8). There were no statistical differences between start and any of the other samples. For the less than 50% group (Figure 2B), there were no statistical differences between any of the samples (n = 10). The relationship between increased uPA activity in the Rem End samples and extent of resection is shown in Figure 2C. There was no statistical correlation below 50% resection (Spearman correlation coefficient = -0.22, *p *= 0.268) but for 50% resection and higher a positive statistical correlation was observed (Spearman correlation coefficient = 0.67, *p *= 0.025, n = 9)

The uPA activity associated with the membrane fractions prepared from samples taken during the operation is shown in Figure 1, for all the patients studied. The activity of the final remnant fraction taken at the end of the operation was increased significantly above the activity of the other fractions. The increased activity of the final remnant fraction was, almost exclusively, confined to those patients who had undergone a resection estimated at 50% or greater (Figure 2A) and there was no increase in those patients in whom the resection was less than 50% (Figure 2B). The percentage change in uPA activity as a function of the resection size for the individual patients is shown in Figure 2C. There was no correlation between uPA activity and the size of the resection below about 50% resection, but a positive correlation was observed when the resection size was 50% or greater.

**Figure 3:**
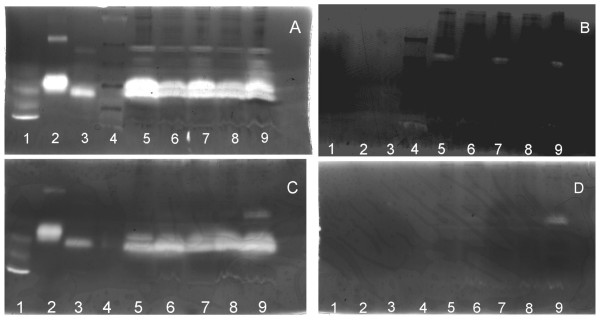
**Zymography gels for patients with greater than 50% resection and less then 50% resection. **Figures 3A and 3B are greater than 50% and Figures 3C and 3D are less than 50%. The gels in Figures 3A and 3C contained plasminogen and the gels in Figures 3B and 3D are the corresponding plasminogen-free control gels. The samples run were, Lane 1- uPA (low molecular weight standard 33 kD, 0.6 ng); Lane 2- tPA standard (65 kD, 1 ng); Lane 3- uPA (high molecular weight standard 54 kD, 0.75 ng); Lane 4–10 μl SeeBlue Plus2 pre-stained standard markers; Lanes 5–9 were washed membrane preparation (30 μg protein / lane); Lane 5 – start; Lane 6 – Res 0; Lane 7 – Rem 0; Lane 8 – Res End; and Lane 9 – Rem End.

Figure 3 shows examples of the zymography gels confirming the presence in the membrane fractions of plasminogen-dependent proteolytic activities. Minor bands in the plasminogen-free control gel (indicating proteolysis which was not plasminogen dependent) were occasionally seen. The activity in these bands was always very much less than the plasminogen dependent activities and plasminogen-free gels had to be more extensively destained in order to visualise these minor bands. Figures 3A and 3B show samples from a patient in whom the estimated resection size was 60% and Figures 3C and 3D show samples from a 15% resection. In both cases, major bands corresponding to high molecular weight uPA and tPA were clearly demonstrable in all the membrane fractions together with minor bands of higher molecular weight. The lanes in plasminogen-free control gel for the patient with the major resection all showed a single very faint band of approximately uniform intensity corresponding to a protein larger than tPA (Figure 3A and 3B). In Figure 3C, a high molecular weight band present only in the lane corresponding to the remnant end sample appeared with approximately equal intensity in the plasminogen-free gel (Figure 3D) indicating that this material was not a plasminogen activator. Although the final remnant sample in Fig 3A had increased uPA activity relative to the other samples, as determined by the fluorometric assay, the major bands of activity in the gel, corresponding to the uPA and tPA markers, showed little change. In our experience, this is a reflection of the qualitative nature of the plasminogen activator zymography. We find that with purified uPA and tPA proteins, an increase of about one order of magnitude is necessary before the bands produced in the gels are notably different.

There were apparent increases in band size associated with the higher molecular weight minor bands in the remnant end sample. Although, at present, the precise nature of these high molecular weight bands is uncertain, similar high molecular weight plasminogen activators have been previously reported [12,13]. The most logical explanation would be an association with either uPAR or plasminogen activator inhibitor type 1 (PAI-1), and on the basis of the present observations such a complex is as likely to contain tPA as uPA. These were not observed in the samples from the patient with a smaller resection. At present, the biological significance of these changes in what appear to be relatively minor bands is uncertain, but there was an association with the large resection size and increased uPA activity, as measured by fluorimetry.

**Figure 4:**
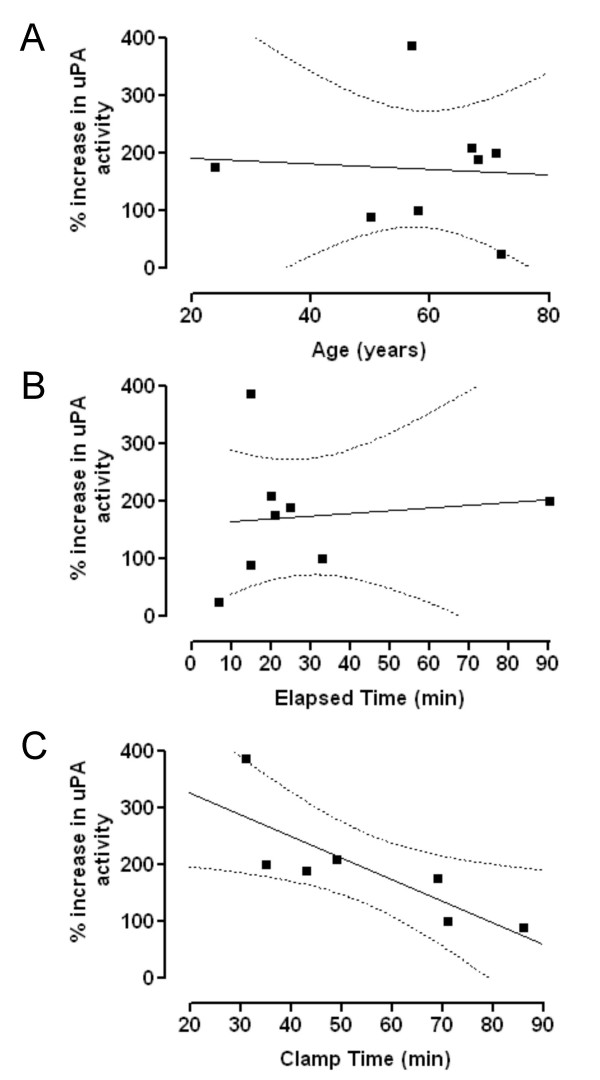
**Relationship between the increase in membrane associated uPA activity (nmol/min/mg protein) and (A) Age, (B) Elapsed time between the time of the resection and the time at which the final sample was obtained, and (C) the total time for which the portal vein was clamped during operation. **Spearman correlation analysis showed no correlation for A (*p *= 0.44, Spearman correlation coefficient = -0.071, n = 8), or B (*p *= 0.27, Spearman correlation coefficient = 0.26, n = 8). In C however, a statistically significant correlation was observed (*p *= 0.012, Spearman correlation coefficient = -0.89, n = 7).

Increases in uPA activity did not correlate with patient age (Figure 4A) or the elapsed time interval between taking the remnant start and the remnant end samples (Figure 4B). There was, however, a statistically significant negative correlation between the increase in activity and the total time for which the blood supply to the liver had been clamped during the operative procedure (Figure 4C), suggesting a link between increased proteolytic activity and hepatic perfusion.

## Discussion

Increased uPA activity and increased levels of uPAR are among the earliest reported events in the remnant liver, following 70% partial hepatectomy in rodents [5]. Since then, several studies have emphasised the importance of the plasminogen system to hepatocyte proliferation and angiogenesis in the regenerating rodent liver [6-9]. We have shown here for the first time in humans that increases in plasminogen activator activity occur following hepatectomy. Increased activity was only seen in remnant liver at the end of the operative procedure when, at least, 15 min had elapsed between the time at which the resection was completed and the last remnant sample taken, and where the magnitude of the resection was estimated to be at least 50% of total liver volume. If, as proposed by Mars *et al. *[5], increased uPA activity is an essential feature at the start of regeneration, then these observations confirm the findings of animal studies that the magnitude of the regenerative response is dependent on the extent of the hepatectomy [14,15]. The present studies suggest that removal of, at least, half the liver mass is necessary to generate the biological signal that results in increased plasminogen activator activity.

The lack of any increase in uPA in response to resections less than 50% compared to the positive correlation between increased uPA activity and increased resection above 50% suggests a threshold event around the 40 to 50% level. The plot shown in Figure 2C bears a striking resemblance to the data in the review by Bucher [14] showing incorporation of tritiated thymidine into DNA following hepatectomy in mature rats. A similar threshold point at about 40% resection, with no correlation below this level and a positive correlation above, was clearly demonstrated in those studies also. The mechanism by which resections greater than about 50% increasingly result in elevated uPA activity and increased DNA synthesis remains elusive. It is still unclear whether the same trigger is responsible for the increases in both systems.

The possibility that the increased uPA activity seen here represents a response to injury rather than an early regenerative response cannot be totally discounted. However, in the rat partial hepatectomy model the anatomy allows removal of the major liver lobe without imposing surgical trauma on the remnant liver suggesting that increased uPA activity is not injury related.

Zymography clearly showed several plasminogen activators to be associated with the membrane fractions. As expected, the major bands corresponded to the high molecular weight uPA and tPA markers. Although uPA and its receptor uPAR have been implicated in the initiation of the liver regeneration process [5,16], no similar role has been ascribed to tPA. The latter binds to both liver endothelial cells (via the mannose receptor) and hepatocytes (by the LDL receptor-related protein) as part of the process by which tPA is rapidly cleared from the circulation by the liver. To date, however, there is no evidence from rodent studies to suggest that binding of tPA to receptors is, in any way, involved in the response to hepatectomy. However, the present study clearly shows tPA activity associated with the liver membrane preparations, and given the ability of tPA to generate active HGF *in vitro *[1] the possibility of a role for tPA in the response of human liver to partial hepatectomy needs to be borne in mind. We also found several minor bands of higher molecular weight, the nature of which is uncertain. These could potentially represent larger forms of the plasminogen activator or the plasminogen activator tightly bound to some other protein. The most likely candidates for such a complex would be uPA associating with either uPAR or PAI-1. The final remnant sample obtained after major resection showed increased amounts of these higher molecular weight components. High molecular weight forms of uPA have been observed in the rat prostate following castration [12] and also in cultured Kaposi sarcoma cells [13]. In the latter, it was suggested that the high molecular weight form of uPA contributed to the characteristic hyperproliferative and invasive phenotype of the Kaposi sarcoma lesions. Increased uPA activity associated with increased metastatic activity seems well accepted and uPA and other members of the urokinase plasminogen activator system (including uPAR and PAI-1) have been selected as novel targets for potential tumour therapies [17]. Whether the high molecular weight forms of uPA are also characteristic of an increased proliferative activity in the liver remains to be fully established.

Presently, the source of the increased uPA activity is uncertain. The very early increase in activity at 1 minute post-hepatectomy in the rat and the lack of any associated increase in mRNA for uPA, precludes any *de novo *protein synthesis [5]. In the present study, the increased activity at 15 minutes post-resection also seems too rapid for a mechanism requiring new protein synthesis. Mars *et al. *[5] suggest that the increased uPA activity seen in rats immediately following partial hepatectomy represents binding of uPA from the blood to the uPA receptor in the liver. The uPAR was undetectable on Western blots from rat liver prior to hepatectomy, but was present in the remnant liver as early as one minute post-hepatectomy and increased in amount during the next 60 minutes. It has been proposed that this increased uPAR binds uPA from the circulating blood resulting in the increased uPA activity within the liver. The suggestion that this is a key element in the initiation of the regeneration process highlights the need for adequate perfusion of the liver. Though the underlying molecular mechanisms remain unclear, interruption of hepatic perfusion generally has adverse effects on the regenerative response [4]. The present study supports the hypothesis that continued liver perfusion is important in the process by which increased uPA activity is generated. Firstly, increases in uPA activity did not occur in the liver that had been resected and removed from the circulation; secondly, for those patients in whom there was an increase in uPA activity, the magnitude of the increase was inversely related to the clamp time, *i.e.*, the longer the liver perfusion was interrupted the smaller the response. Thus, in the present study, increased uPA activity was negatively correlated with total clamp time suggesting that hypoxia, which has been shown to induce uPAR expression in cells in culture [18-20], is not a likely mediator of the uPA increase seen here.

Finally, despite the proliferative capacity of hepatocytes and the ability of the liver to regenerate declining with age [14,15], we found no correlation between age and basal uPA activity and the increase in remnant liver uPA activity was also not age dependent.

## Conclusions

In the present paper, we show early increases in uPA activity can be demonstrated in the remnant liver following resection of metastatic tumours in patients in whom the resection was estimated to be 50% or greater. To the best of our knowledge this is the first time this has been demonstrated. Such increases are amongst the earliest events following hepatectomy in rats, where they are considered to initiate changes in the extracellular matrix essential for subsequent hepatocyte division. Thus, our results support a similar role in the initiation of liver regeneration in man.

## Methods

### Patients

The South Sheffield Research Ethics Committee approved the research protocol and fully informed consent was obtained. Eighteen patients undergoing partial hepatectomy for the removal of hepatic metastases secondary to primary colonic tumours were studied. There were 7 males and 11 females with an age range from 24 to 78 years (median 67.5).

### Operative Procedure

Standard operative procedures were followed. The liver was mobilised and the resection delineated with diathermy. The portal inflow was clamped while resection with an ultrasonic dissector was carried out. Typically, the portal inflow was released every 15 minutes for 5 minutes intervals to prevent ischaemic damage and the total clamping time was recorded. Resection margins were sent separately for histopathology. The magnitude of the resection was estimated as percentage of the total liver volume, by the surgeon.

The following samples were taken from tumour-free regions of the liver during the operation. A sample was obtained before the resection was started (this was labelled 'Start'). Immediately following resection samples were taken from the remnant liver (labelled 'Rem 0' for remnant liver at time 0) and from the resected liver as far away from the tumour as possible (labelled 'Res 0' for resected liver at time 0). The samples were placed in cryovials and immediately frozen in liquid nitrogen in the operating theatre. A second sample of the resected liver (labelled 'Res end') was kept at room temperature until the end of the operative procedure and was only transferred to liquid nitrogen when the final sample from the remnant liver was taken. The final sample ('Rem end' for remnant end) was taken from the remnant liver as late into the operation as possible and frozen immediately. The 'Res end' sample was also frozen at this time. The interval between the time of sampling 'Rem 0' and 'Rem end' ranged from 7 to 90 minutes. The median was 20.5 minutes and 15 of the 18 intervals were between 10 and 33 minutes. Samples were stored in liquid nitrogen in the laboratory and only thawed immediately prior to analysis.

### Materials

Casein, plasminogen, uPA (high and low molecular weight forms) and tPA were purchased from Calbiochem (CN Biosciences Ltd., UK). The fluorometric substrates 7-amino-4-methylcoumarin (AMC), Z-Gly-Gly-Arg-AMC and EGR-CMK (Glu-Gly-Arg-Chloromethylketone) were from Bachem Ltd. (UK). Electrophoresis reagents were from BioRad and Geneflow Ltd. Other reagents were from Sigma-Aldrich Co Ltd. (Poole, UK).

### Sample preparation

Liver samples were homogenised in a ten-fold volume of homogenisation buffer: 250 mM sucrose / 10 mM MOPS pH 7.4 containing the protease inhibitors E-64 (20 μM), Pepstatin A (20 μM), and EDTA (0.2 mM). Inhibitors against the serine proteases, which include uPA, were not included.

### Membrane preparation

A membrane preparation was made by differential centrifugation of the homogenate in a TLS 55 swinging bucket rotor in a Beckman TL-100 bench top ultracentrifuge (Beckman Coulter Ltd., High Wycombe, UK). The homogenate was initially centrifuged at 40,000 g, for 20 minutes, to pellet large cell organelles such as nuclei and mitochondria. After centrifugation the fat at the top of each tube was removed with a piece of tissue and the supernatants transferred to clean tubes and recentrifuged at 105,000 g, for 1 hour. The membranous pellets were then washed twice by resuspending in homogenisation buffer and recentrifuged at 105,000 g, for 1 hour. All centrifugations were carried out at 4°C.

The protein content of the homogenates and membrane preparations was determined by the BCA (bicinchoninic acid) method [21] using a kit from Sigma-Aldrich.

### uPA fluorometric assay

uPA activity was determined by a fluorimetric continuous rate assay of Z-Gly-Gly-Arg-AMC hydrolysis using a Perkin Elmer LS50B fluorimeter linked to an IBM compatible computer running the FLUSYS software [22]. Cleavage at the Gly-Arg bond by uPA releases the AMC from its quenched state [23] and the rate at which fluorescence is produced taken as a measure of uPA activity.

At the end of the assay, EGR-CMK (Glu-Gly-Arg-Chloromethylketone), a chemical inhibitor of uPA, was added to check that this compound inhibited the measured activity. Any activity still persisting was taken as not uPA-mediated and subtracted from the rate measured in the absence of EGR-CMK.

Since the biologically relevant fraction of uPA is generally considered to be associated with its receptor uPAR and therefore localised to the cell membrane, uPA activity measurements were performed with washed membrane preparations rather than with total liver homogenates. Preliminary experiments demonstrated the necessary linear response between the measured activity and the volume of membrane preparation assayed (data not shown).

All samples were assayed in triplicate at two sample volumes to ensure linearity of activity with amount of extract. The measured rates were then adjusted for the protein concentration of each sample to give a rate in nmoles/min/mg of protein.

### Zymography

Zymography was carried out with 7.5% SDS PAGE two-substrate gels essentially as described by Bryson *et al. *[24]. The control, plasminogen-free, gels contained casein alone (final concentration of 6 mg/ml gel) and the test gels contained plasminogen at a final concentration of 9.3 μg (1.12 U) / ml gel in addition to the casein. Following electrophoresis gels were washed in 25% (v/v) Triton X-100, for 1 hour at room temperature, and then in 50 mM Tris (pH 7.6) for 16–20 hours and at 37°C, prior to staining with Coomassie blue. Purified uPA (high molecular weight and low molecular weight) and tPA (all from CalBiochem, CN Biosciences Ltd., Nottingham UK) and SeeBlue Plus2 Pre-stained standards (Invitrogen Life Technologies, Paisley, UK) were included on each gel as markers.

### Graph plotting and statistical analysis

All Figures were generated and analysed with the GraphPad Prism package (version 3.0). Statistical analyses (Student's *t *tests, simple linear regression, Spearman correlations) were performed using the software in the cited package.

## Authors Contributions

DM initiated the study, carried out the zymography experiments, prepared tissue extracts and drafted the manuscript. KS prepared tissue extracts and carried out the fluorometric assays. AWM and NCB participated in the design and coordination of the study. All authors have read and approved the final manuscript.
